# Neurological updates: neurological complications of CAR-T therapy

**DOI:** 10.1007/s00415-020-10237-3

**Published:** 2020-11-02

**Authors:** Emma C. Tallantyre, Nia A. Evans, Jack Parry-Jones, Matt P. G. Morgan, Ceri H. Jones, Wendy Ingram

**Affiliations:** 1 School of Medicine, Cardiff University, Cardiff, UK; 2 Department of Neurology, Cardiff and Vale University Health Board, Cardiff, UK; 3 Department of Pharmacy, Cardiff and Vale University Health Board, Cardiff, UK; 4 Midlands and Wales Advanced Therapy Treatment Centre, Birmingham, UK; 5 Department of Critical Care, Cardiff and Vale University Health Board, Cardiff, UK; 6 Department of Haematology, Cardiff and Vale University Health Board, Cardiff, UK

**Keywords:** Neurological, Chimeric antigen receptor T cell (CAR-T), Adverse events, Side-effects

## Abstract

Chimeric antigen receptor (CAR)-expressing T cells now offer an effective treatment option for people with previously refractory B cell malignancies and are under development for a wide range of other tumours. However, neurological toxicity is a common complication of CAR-T cell therapy, seen in over 50% of recipients in some cohorts. Since 2018, the term immune effector cell-associated neurotoxicity syndrome (ICANS) has been used to describe and grade neurotoxicity seen after CAR-T cells and other similar therapies. ICANS following CAR-T therapy is usually self-limiting but can necessitate admission to the intensive care unit and is rarely fatal. As CAR-T therapies enter routine clinical practice, it is important for neurologists to be aware of the nature of neurological complications. Here, we summarise the clinical manifestations, mechanisms, investigations and recommended treatment of CAR-T-related neurotoxicity, focusing on the licensed CD19 products.

## Introduction

**Fig. 1:**
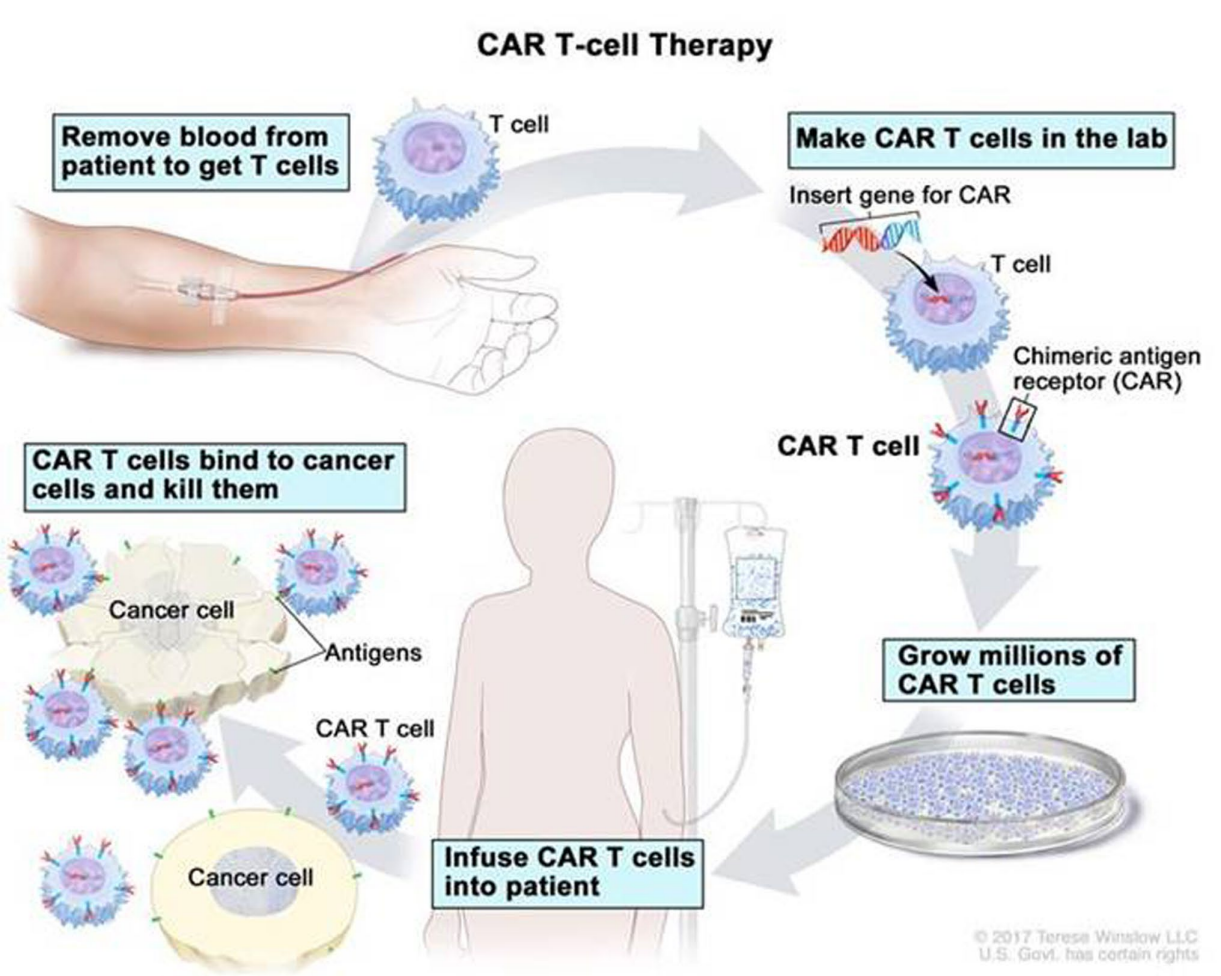
Schematic diagram illustrating the process of CAR T cell therapy © 2017 Terese Winslow LLC, US Government has certain rights. Note for the editor: copyright has been granted for re-use of this image. I can supply the agreement

The development of chimeric antigen receptor (CAR)-expressing T cells represents a major advance for the treatment of haematological malignancy. Autologous or allogeneic T cells are leukapheresed and genetically modified ex vivo by viral transduction to generate an advanced therapy medicinal product (ATMP; Fig. 1). The T cells are modified to express a CAR, which features an antigen detecting single-chain variable fragment, expressed on the cell surface acting as the target binding domain. This extracellular antigen recognition moiety is fused via a transmembrane domain to an intracellular co-stimulatory domain (such as CD28 and 4-1BB) and a CD3-zeta activation domain. The transduced anti-tumour CAR-T cells are expanded ex vivo and infused into the patient, following lymphocyte depleting chemotherapy to facilitate CAR-T cell expansion in the host. The intrinsic activation mechanism within CAR-T cells allows them to become fully activated and acquire the full repertoire of effector functions on encountering tumour antigen, without the need for major histocompatibility complex-epitope presentation.

Autologous anti-CD19 CAR-T cells are the most widely studied. Clinical trials demonstrating response rates of 50–80% in previously refractory B cell lymphomas [1, 2], and 65–90% in acute lymphoblastic leukaemia (ALL) [3], have led to the licensing of two CAR-T products. Tisagenlecleucel (Kymriah) is licensed by the European Medicines Agency for treatment of refractory B cell ALL in children and young adults up to 25 years of age and refractory diffuse large B cell lymphoma (DLBCL) in adults. Axicabtagene ciloleucel (Yescarta) is licensed for the treatment of adults with refractory DLBCL or primary mediastinal large B cell lymphoma. Approval for a third product, lisocabtagene maraleucel (Lisocel or JCAR017), is expected soon [4]. The existing anti-CD19 CAR-T therapies are currently being investigated for use earlier in the treatment pathway, and for expanded indications. New CAR-T therapies are also being developed for solid tumours including gastrointestinal cancer, skin cancer, genitourinary, breast, gynaecological, lung, multiple myeloma, brain tumours and sarcoma [5, 6].

**Fig. 2:**
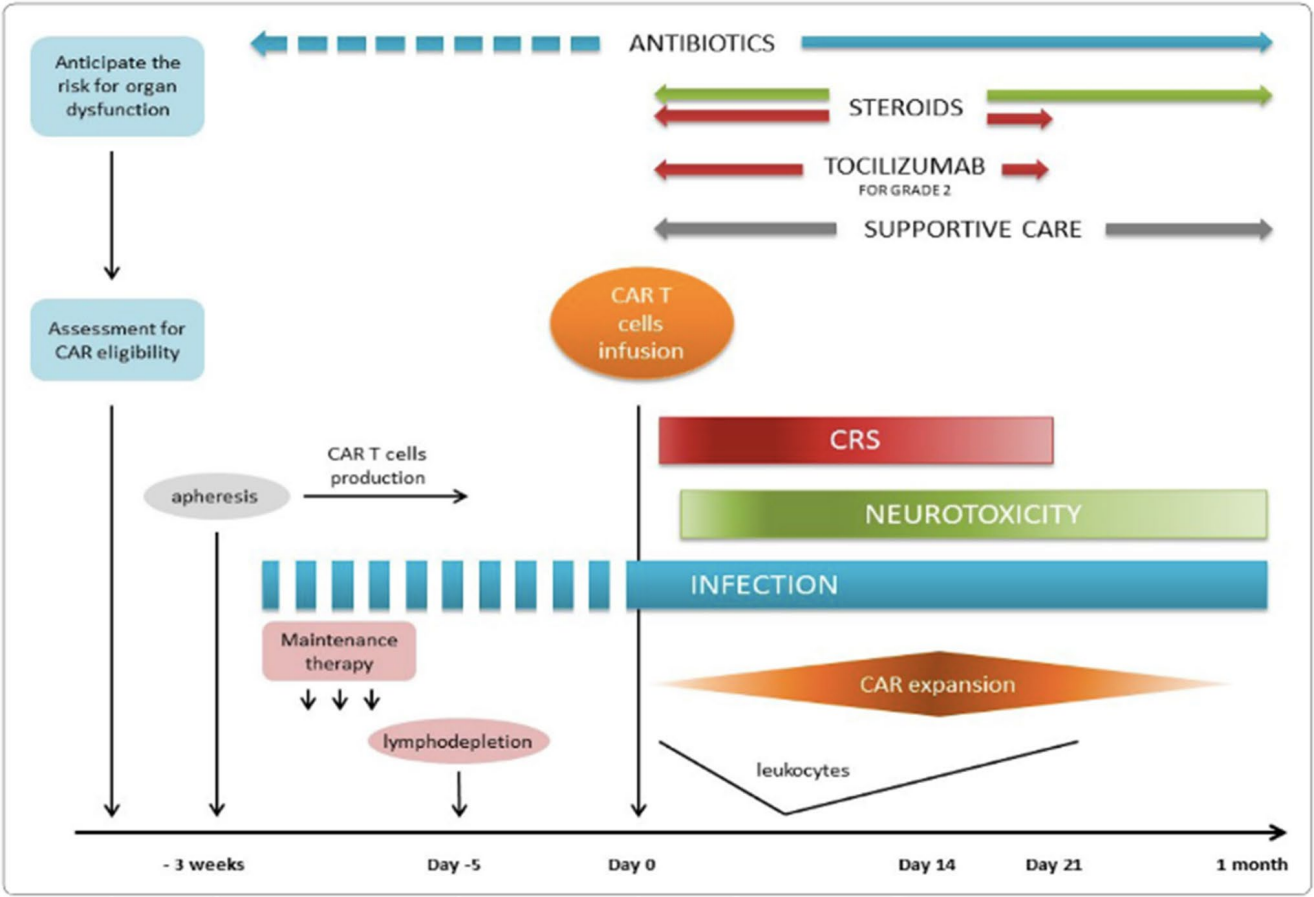
Timing of complications following CAR T cell infusion (from [47], with kind permission Copyright© 2020, Springer Nature)

Despite their impressive efficacy, anti-CD19 CAR-T therapies are associated with high rates of toxicities, the most common being cytokine release syndrome (CRS) and neurotoxicity (Fig. 2). CRS is a multi-system clinical syndrome with manifestations ranging from mild flu-like symptoms to life-threatening inflammatory response. Features can include fever, tachycardia, hypotension, hypoxia, and organ dysfunction. CRS arises due to profound and generalised immune system activation, associated with supra-physiological levels of circulating cytokines. Symptomatic management with antipyretics, fluids, oxygen therapy and organ support, including advanced airway support where necessary, form the basis of supportive care. Tocilizumab, an interleukin-6 (IL-6) receptor blocking monoclonal antibody, is used for more severe cases and often results in rapid and complete resolution of CRS [7]. Tocilizumab offers an opportune mechanism of action by blocking a key driver for CRS, but with little impact on T cell function.

Neurotoxicity following CAR-T therapy is usually self-limiting but can necessitate admission to the intensive care unit (ICU) and is rarely fatal. As CAR-T therapies enter routine clinical practice, it is important for neurologists to be aware of the nature of neurological complications. Here, we summarise the clinical manifestations, mechanisms, investigations and recommended treatment of CAR-T-related neurotoxicity, focusing on the licensed CD19 products.

## Clinical features of neurotoxicity

A diverse range of clinical manifestations of neurotoxicity have been reported. This diversity may be partly explained by the varied nomenclature used to describe the hallmark feature of encephalopathy. Furthermore, there may be some variability in the presentation of neurotoxicity with different CAR products. However, many individuals with neurotoxicity seem to demonstrate a stereotypic pattern and temporal evolution of symptoms [5, 8].

Common early clinical manifestations of neurotoxicity include tremor and mild problems with attention and expressive language [8–11]. Tremor tends to be heightened physiological tremor, but more disabling rest, postural or intention tremors have been described [8]. Dysgraphia and expressive dysphasia are common, especially difficulty naming [5, 12]. Other features of the mild encephalopathy include disorientation to time and place, short-term memory dysfunction, apraxia, hallucinations and behaviour disturbance including impulsivity, emotional lability or abulia [8, 13]. Visual symptoms are reported but tend to be mild and transient, often with migrainous characteristics [8, 10]. Lethargy and headache are frequent, but relatively non-specific, early symptoms [5, 9, 11]. Patients can be somnolent in the early stages but often there is preserved alertness [8, 11, 13].

The majority of cases of neurotoxicity are mild or moderate [8]. Those who do progress to severe neurotoxicity, usually do so over hours to days, and follow a reverse pattern during recovery [5, 8]. Occasionally, neurotoxicity presents with rapid onset encephalopathy and features of cerebral oedema that rapidly proves fatal. This fulminant presentation, in the absence of antecedent clinical signs, may have a distinct pathophysiology from the more characteristic, reversible neurotoxicity [5, 13]. As neurotoxicity evolves to become more severe, expressive language function often deteriorates, including impaired naming, paraphasic errors, verbal perseveration or, in extreme cases, global aphasia or mutism [8, 13]. Myoclonus, ataxia, meningism and autonomic instability may become apparent [5, 8, 13]. There may be mild somnolence or a more depressed level of consciousness requiring ICU support [11]. Paradoxically, some individuals may manifest with an agitated delirium [8]. Subclinical electrographic or clinical seizures often follow the development of severe expressive dysphasia. Seizures tend to be generalised tonic–clonic seizures, with evidence of focal onset in some cases [11, 13]. Some authors consider seizures to be more likely in children and in those with radiographic evidence of cerebral oedema [10]. Seizures can be prolonged despite early administration of seizure rescue medications [14].

Focal weakness of the limbs or face, or sudden onset dysphasia, is seen in a significant minority of cases [8–11, 13]. Some cases mimic stroke or seizure-like events, in the absence of MRI or EEG correlates [13]. Pre-existing vascular risk factors appear to be more common in this group and deficits are typically transient [8].

Examination of people with severe neurotoxicity can reveal frontal release signs such as palmomental, snout, or grasp reflexes. There may be increased tone, evidence of focal weakness or facial automatisms [8, 13].

## Timing and relationship with CRS

Neurotoxicity can occur in absence of CRS [9], but severe neurotoxicity almost always occurs in people who experienced preceding CRS [8, 10, 13]. Some authors believe neurotoxicity has a biphasic presentation; the first phase coinciding with CRS symptoms, tending to be milder and shorter duration, and a second phase occurring after the fever and other CRS symptoms subside, and tending to be more severe and protracted [12]. Cases of delayed neurotoxicity have been reported, with seizures or confusion occurring 3 or 4 weeks after CAR-T cell therapy [12].

On average, the symptoms of neurotoxicity start later than those of CRS (Fig. 2) [9, 13]. Median time from CAR-T infusion to onset of neurotoxicity is reported to be 4–6 days [2, 8, 11, 13, 15]. Peak neurotoxicity is reported to occur around day 7–9 with a median duration of 5–13 days [8, 11–13, 15].

The majority of cases of neurotoxicity resolve within 3–8 weeks [16]. However, up to 10% of cases of neurotoxicity were unresolved at the final follow-up visit of the seminal clinical trials (median follow-up 3–28 months) [2, 16–18]. Detailed long-term follow-up data are so far scarce. There have been anecdotal reports of long-term sequelae of neurotoxicity including epilepsy [11], and mild memory impairment [15]. Twelve-month follow-up data on 86 people who received CD19 CAR-T cells for relapsed/refractory ALL, NHL, or CLL during phase I/II clinical trials found no neurological or psychiatric adverse events persisting beyond 90 days. However, there has been a recent report of an unusually delayed and recurrent case of ICANS following axicabtagene ciloleucel administration for DLBCL. In the 76-year-old woman, ICANS occurred at day 6 post CAR-T infusion, responding initially to dexamethasone but recurring after steroid taper, and recurring again almost 6 months post infusion, proving steroid refractory on the last occasion [19].

## Definition and grading of CAR-T-associated neurotoxicity

When CAR-T therapies were developed, the widely used Common Terminology Criteria for Adverse Events (CTCAE) scoring system were used to grade neurotoxicity [20]. The generic CTCAE system assigns a grade between 1 and 5 for mild, moderate, severe, life-threatening or fatal events. However, the CTCAE system includes several terms relevant to encephalopathy, which overlap. In 2018, the CAR-T cell-therapy-associated TOXicity (CARTOX) working group published recommendations for the grading of neurotoxicity [12], which was refined in 2019 by the American Society for Transplantation and Cellular Therapy (ASTCT) [5]. The ASTCT group coined the term immune effector cell-associated neurotoxicity syndrome (ICANS). The refined definition and grading system were aimed at including a wide range of clinical features, but omitting those with poor specificity or confounding causes, and to acknowledge that similar syndromes can be seen with different immune treatments. The ICANS grading system remains in widespread use (Table 1) [5], assigning a grade between 1 and 4, according to scores in five domains. First, the Immune Effector Cell-Associated Encephalopathy (ICE) is used to assess cognition. The ICE is a 10-point score that draws upon components of the Mini-Mental State Examination (MMSE) to assess orientation, naming, ability to follow commands, handwriting and attention. The other four domains in the ICANS grading system are: level of consciousness, seizures, motor findings and evidence of elevated intracranial pressure. A separate but similar grading scale was developed for children < 12 years [5].

**Table 1:** Immune Effector Cell-Associated Encephalopathy (ICE) grading score of ICANS (adapted from [5], with kind permission, © 2018 American Society for Blood and Marrow Transplantation)

ICE score
Orientation: orientation to year, month, city, hospital: 4 points Naming: ability to name three objects (e.g. point to clock, pen, button): 3 points Following commands: ability to follow simple commands (e.g. “Show me 2 fingers” or “Close your eyes and stick out your tongue”): 1 point Writing: ability to write a standard sentence (e.g. “Our national bird is the bald eagle”): 1 point Attention: ability to count backwards from 100 by 10: 1 point
Scoring: 10, no impairment 7–9, grade 1 ICANS 3–6, grade 2 ICANS 0–2, grade 3 ICANS 0 due to patient unarousable and unable to perform ICE assessment, grade 4 ICANS

## Incidence and severity of neurotoxicity

Despite recent changes to the definition and grading of neurotoxicity, published clinical trial and cohort data for the licensed CD19 CAR-T therapies used the CTCAE grading system. Some trials chose to count any seizure as grade 3 neurotoxicity [2, 21], and one trial counted any motor weakness as grade 4 neurotoxicity [2]. Interestingly, the CTCAE system seems to over-report neurotoxicity; re-analysis of safety data graded using the ICANS system resulted in downgrading over 50% of neurotoxicity events in one trial to grade zero [22].

During clinical trials of the licensed CD19 CAR-T products, the overall incidence of neurotoxicity using the CTCAE grading ranged from 21 to 64%, and incidence of severe neurotoxicity was 5–50% [13, 23–32]. In a cohort of 43 children and young adults with ALL, neurotoxicity was observed in 44% cases (21% severe) [14]. Whereas a further study of 100 adults with a range of (predominantly B cell) malignancies, neurotoxicity was reported in 48% cases (20% severe) [8].

## Risk factors for ICANS

The wide variation in the reported incidence of neurotoxicity may be explained by the factors relating to the underlying disease, prior treatments, the pre-morbid health of the recipient, as well as the features relating to the CAR-T construct and manufacture.

Higher pre-treatment B cell tumour burden appears to predict higher likelihood of neurotoxicity following CD19 CAR-T [2, 9, 13, 16, 33]. Rates of ICANS may vary according to the type of B cell malignancy [11]. Higher CAR-T dose, and certain conditioning regimens may predict the risk of subsequent neurotoxicity [11]. In particular, use of fludarabine for lymphodepletion may predispose to ICANS, possibly as a result of greater in vivo CAR-T cell expansion, or as a direct toxic effect of fludarabine on the brain or endothelium [11, 18]. ICANS is also predicted by the presence of earlier and higher peaks in serum CRP, serum cytokines, and symptoms of CRS including high fever following CD19 CAR-T treatment [2, 8, 11, 13, 14]. The presence of pre-morbid neurological disease or MRI brain changes also appears to increase the risk of ICANS, which has led to a cautious use of CAR-T therapy in patients with known CNS involvement of leukaemia or lymphoma [11, 14].

Features of the CAR design are also likely to be relevant in predicting the incidence of toxicity [10, 34, 35]. In particular, the use of a humanised or murine single-chain variable fragment (scFv), the use of hinge and transmembrane domains derived from either the CD28 or the CD8α molecule, and the use of CD28 co-stimulatory moieties have all been postulated to be relevant to the likelihood of ICANS [35, 36]. The currently licensed anti-CD19 CARs have scFvs derived from murine antibodies. In a recent phase 1 trial of a new CD19 CAR-T therapy, featuring a fully humanised scFv and a hinge and transmembrane domain derived from CD8α, the anti-lymphoma effect appeared to rival licensed CD19 CAR-T products, but ICANS was only observed in 1 out of 20 (5%) recipients [37].

The CAR manufacturing approach may also be relevant. A trial using a CAR construct, that was already in existence (CD19 CAR with a CD28 co-stimulatory domain) but using a different manufacturing approach, was closed early because of a high incidence of fatal ICANS [38].

Overall, the rates of ICANS appear lower for non-CD19 CAR-T cells-targeting hematologic malignancies, and neurotoxicity has not been reported in solid tumour CAR-T therapies (with exception of brain tumour trials) [10]. It seems likely that the higher risk of ICANS after CD19-targeted therapies reflects their capacity for robust T cell activation compared to T cell therapies targeting other antigens expressed on different tumours, possibly related to the higher accessibility to CD19 tumour cells, or their level of antigen expression [11].

## Potential mechanism of neurotoxicity

The pathophysiology of ICANS following CAR-T therapy is not fully understood, although several possible mechanisms have been proposed. The heterogeneity in clinical presentations, imaging and laboratory features of ICANS make it plausible that different mechanisms are at play [10]. Furthermore, confounding contributors of organ dysfunction, hypoxaemia and infection may lead to a wider range of presentations [12].

The finding that high tumour burden, high levels of CAR-T expansion and severe CRS all predict subsequent ICANS, supports the concept that cytokine release is likely to be relevant. Indeed, several groups have shown blood cytokine levels to be higher in people with ICANS versus those without. Numerous groups have also demonstrated high cerebrospinal fluid (CSF) levels of cytokines in people with ICANS [11, 13, 14, 39], but uncertainty remains over whether cytokines are peripherally produced and trafficked into the CNS, or centrally produced. Evidence of endothelial activation and blood brain barrier (BBB) disruption in people with ICANS is well established, raising the possibility that peripheral cytokines cross into the CNS [11]. However, CAR-T cells have also been demonstrated to gain access to the CNS, and are present in higher numbers in the CSF of those with ICANS [40]. Some groups have reported disproportionately high levels of ﻿cytokines in CSF (versus blood) in severe ICANS, supporting at least some CNS cytokine production [13, 39].

Cytokine profiling of the blood and CSF in the context of ICANS has shown a range of candidate molecules. The majority report high IL-6, the cytokine most strongly associated with CRS [8, 10, 13, 14, 39]. One group observed that the rate of rise in serum cytokine concentration, as well as the peak concentration was predictive of ICANS [11]. Severe ICANS is also associated with elevated levels of a range of other cytokine or chemokines including IL-1Ra, IL-2Ra, IL-1, IL2, IL-8, IL-10, IL-15, IFN-gamma, CCL-2, granzyme B, GM-CSF and VEGF [2, 14, 39]. This suggests that cells other than T cells, including endothelial cells and monocytes, may be implicated [10, 41]. The role of macrophage activation in ICANS is further supported by markedly elevated serum ferritin in some cases, reminiscent of macrophage activation syndrome and hemophagocytic lymphohistiocytosis [42]. Neuropathologic studies at autopsy after fatal ICANS have demonstrated a widespread inflammatory process in some cases, involving dense macrophage infiltration of white matter, numerous microglial cells, and a moderate CD8+ T cell infiltrate [18, 39], and CAR-T cells within the brain tissue [11, 39]. In other cases, evidence of oedema, astrocytic damage, activated microglia and BBB dysfunction have been present in the absence of a lymphocytic infiltrate [8, 10, 43].

The post mortem and CSF findings demonstrating lymphocytosis raise the possibility that ICANS arises as a result of a T cell encephalitis. Post mortem cases have not shown any evidence of herpes simplex virus 1 or 2, cytomegalovirus, varicella zoster virus, JC virus, adenovirus, or Epstein–Barr virus [18]. Reactivity of CAR-T cells with a non-target antigen in the brain would be a plausible mechanism of encephalitis, although it remains unclear whether CNS involvement of leukaemia predicts a higher risk of ICANS [9]. Furthermore, the low incidence of brain parenchymal abnormality on MRI in people with ICANS does not necessarily align with the presence of encephalitis.

It is possible that endothelial activation and BBB permeability is of fundamental importance in ICANS, allowing cytokines and CAR-T cells pass into the CNS [11]. It has been proposed that a tendency towards endothelial activation may even pre-date CAR-T infusion in those who go on to develop ICANS [11], implying certain individuals may be at risk. In susceptible individuals, a more permeable BBB may expose brain vascular pericytes to high concentrations of circulating cytokines, inducing further endothelium-activation [11]. This process is somewhat analogous to eclampsia or posterior reversible encephalopathy syndrome (PRES) [8]. Once cytokines have access to the CNS they may exert their effects in the absence of a centrally directed inflammatory response. For instance, one group found high levels of quinolinic acid and glutamate, agonists to *N*-methyl-*d*-aspartate (NMDA) receptor, in CSF during ICANS, suggesting that endogenous excitatory agonists may be involved [13]. Raised CSF levels of GFAP, S100b during ICANS also raise the possibility that astrocytic injury may contribute to osmotic dysregulation and cerebral oedema.[14]

## Investigation of neurotoxicity

In a patient with suspected ICANS, investigations are mostly aimed at ruling out mimics or contributory factors including infection, cerebral involvement of B cell malignancy, drug toxicity, metabolic derangement or organ dysfunction. CT and MR imaging of the brain is normal in most cases of mild ICANS and in many individuals with more severe ICANS [8, 10, 13]. CT imaging is often more practical in an unwell patient and is useful in excluding pathology such as cerebral haemorrhage, infarct and oedema. Where MRI is abnormal, various patterns have been observed. In some cases, there are T2/FLAIR hyperintensities involving the bilateral thalami and brainstem, or cerebral white matter, without diffusion restriction, which resolve on subsequent imaging performed after neurologic symptom resolution [13, 14]. Another MRI pattern observed is transient T2 hyperintense lesions within the splenium of the corpus callosum or in a gyral pattern that not only restricts on diffusion imaging but also resolves on follow-up imaging [10, 13]. Two cases of primary cerebellar involvement have been reported, although one had previous radiation-related cerebellar injury [14]. A small number of cases of ischaemic stroke and subarachnoid haemorrhage have been reported, as well as a single case of bilateral intra-labyrinthine cochlear haemorrhage [8]. Where MR imaging has been normal, occasionally focal neurological deficits have been associated with FDG-PET hypometabolism, or elevated velocities on transcranial Doppler ultrasound [8].

Electroencephalogram (EEG) most commonly shows frontal intermittent rhythmic delta activity, loss of posterior dominant rhythm and diffuse or focal slowing with or without triphasic waves, in keeping with an encephalopathic state [8, 10, 12, 13]. Most often, EEG seizure activity coincides with clinical seizure activity, but non-convulsive status may occur in as many as 10% of cases [10, 12, 13]. The role of continuous EEG monitoring remains uncertain. Real-time monitoring may be available on some, but not all ICUs. However, real-time interpretation of continuous EEG presents a challenge in many departments. The role of continuous EEG may remain limited to the monitoring of seizure control of those with proven status epilepticus, although further evidence may support a wider role in people with ICANS.

Lumbar puncture may be challenging in an encephalopathic patient and CSF findings diagnostic of ICANS are lacking. While CSF can show evidence of BBB permeability and/or lymphocytic pleocytosis in an individual with ICANS, both are non-specific and neither correlate with neurotoxicity severity [8, 13]. CSF examination is mainly useful to rule out concurrent infection, using directed antimicrobial testing, and to rule out CNS lymphoma or leukaemia. Serum and CSF biomarkers diagnostic of ICANS are highly sought after but none are yet validated for use in clinical practice.

## Management of neurotoxicity

**Fig. 3:**
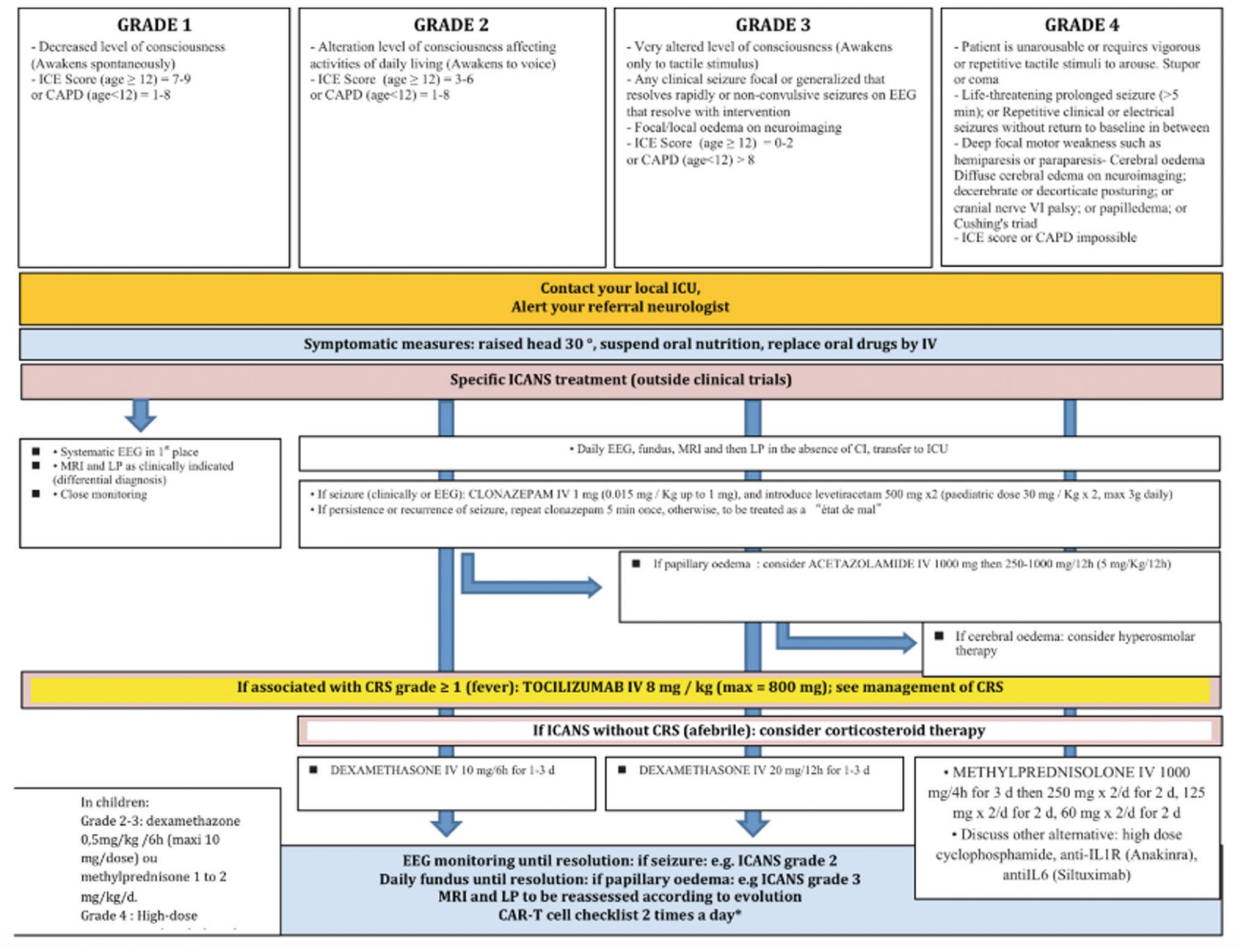
Best practice recommendations for the management of ICANS following CAR T cell therapy according to the European Society for Blood and Marrow Transplantation (EBMT) and the Joint Accreditation Committee of ISCT and EBMT (JACIE). From [45], with kind permission Copyright© 2020 Ferrata Storti Foundation

Supportive management of neurological complications of CAR-T therapy requires close liaison and partnership between haematology, neurology and critical care colleagues. Regular morbidity and mortality review of all cases where neurological complications have occurred is valuable to build local expertise. Systematic monitoring of patients following CAR-T cell infusion should be performed on a haematology ward. Clear care algorithms should be in place with appropriate triggers for referral should complications arise, that are reviewed regularly. Vigilant management of infection or metabolic derangement is important (Fig. 3). Neurological assessment is recommended prior to CAR-T therapy and daily during the first 10 days following CAR-T cell infusion [44]. The patient may be transitioned to management through an ambulatory care setting following day 10 providing there are no active toxicities. Such patients are recommended to remain within 1 h of the treatment centre for the first 28 days after CAR-T treatment, with continuous presence of a caregiver who is trained in the recognition of symptoms including ICANS [44]. The ICE score (Table 1) and ICANS grading system (Table 2) are the most widely used tools to detect and monitor ICANS, but in our experience may lack sensitivity in early cases.

**Table 2:** Grading of immune effector cell-associated neurotoxicity syndrome (ICANS) by American Society for Transplantation and Cellular Therapy (ASTCT) (adapted from [5], with kind permission, © 2018 American Society for Blood and Marrow Transplantation)

Neurotoxicity domain	Grade 1	Grade 2	Grade 3	Grade 4
ICE score*	7–9	3–6	0–2	0 (patient is unrousable and unable to perform ICE)
Level of consciousness	Awake spontaneously	Awakens to voice	Awakens only to tactile stimuli	Patient is unrousable or requires vigorous tactile stimuli to arouse. Stupor or coma
Seizure	N/a	N/a	Any clinical seizure, focal or generalised, that resolves rapidly. Or non-convulsive seizures on EEG that resolve with intervention	Life threatening prolonged seizures (> 5 min), or repetitive clinical or electrical seizures without return to baseline in between
Motor findings	N/a	N/a	N/a	Deep focal motor weakness such as hemiparesis or paraparesis
Elevated ICP/cerebral oedema	N/a	N/a	Focal/local oedema on neuroimaging	Diffuse cerebral oedema on neuroimaging; decerebrate or decorticate posturing; or cranial nerve palsy; or papilloedema; or Cushing’s triad

ICANS grade is determined by the most severe event (ICE score, level of consciousness, seizure, motor findings, raised ICP/cerebral oedema) not attributable to any other cause; for example, a patient with an ICE score of 3 who has a generalised seizure is classified as grade 3 ICANS

ICANS if unarousable. Depressed level of consciousness should be attributable to no other cause (e.g. no sedating medication). Tremors and myoclonus associated with immune effector cell therapies may be graded according to CTCAE v5.0, but they do not influence ICANS grading. Intracranial haemorrhage with or without associated oedema is not considered a neurotoxicity feature and is excluded from ICANS grading. It may be graded according to CTCAE v5.0

*N/A* not applicable

*A patient with an ICE score of 0 may be classified as grade 3 ICANS if awake with global aphasia, but a patient with an ICE score of 0 may be classified as grade 4

Corticosteroids are the mainstay of treatment for ICANS. The lymphotoxic action of corticosteroids poses a risk of reducing the anti-malignancy effect of CD19 CAR-T cells [9, 45], but the efficacy in reversing ICANS makes them appropriate therapy for moderate or severe ICANS. Where conscious level is depressed, dexamethasone 10 mg qds for 1–3 days is recommended. In grade 4 ICANS where patients may be unrousable, have status epilepticus or imaging features of cerebral oedema, then methylprednisolone 1000 mg is recommended [44, 46].

Tocilizumab is a humanised monoclonal antibody that binds to both soluble and membrane bound IL-6 receptor (IL-6R). It was developed to treat rheumatological disorders, but can be used for ICANS, although evidence suggests the benefit is most when ICANS occurs early and/or in combination with CRS [10, 12]. This may relate to increased permeability of the BBB at an early stage, facilitating greater access for tocilizumab into the CNS [12]. In cases of isolated ICANS, tocilizumab may not benefit, and there have been some concerns that it could in fact paradoxically increase CNS IL-6 levels, potentially aggravating ICANS [14, 16], This has led some treatment algorithms to recommend avoiding use of tocilizumab even when it coincides with CRS [46]. Siltuximab, which directly binds IL-6, may be more beneficial in cases of isolated ICANS, but it has mainly been trialled where tocilizumab has failed, which may be too late [10]. Preclinical work suggests future targeting of IL-1, using therapies such as anakinra (monoclonal antibody to IL-1R) may benefit ICANS, although clinical evidence remains anecdotal [16, 41, 46]. Even direct targeting of monocytes, rather than eliminating their downstream targets may warrant consideration [16].

Anti-seizure medications were introduced prophylactically as ICANS emerged during early trials. However, opinion remains divided about the benefit of prophylactic use of anti-epileptics as they have not been clearly demonstrated to reduce seizure complications [10, 13, 14]. Treatment of emergent seizures with standard benzodiazepine and antiepileptic drug titration appears effective in most cases although refractory or prolonged seizures can occur [13, 14]. Levetiracetam appears the favoured choice of antiepileptic in people with ICANS, likely due to its low incidence of drug–drug interactions, low risk of cardiotoxicity, and favourable safety profile in people with hepatic dysfunction [10, 12].

Patients with grade > / = 3 ICANS should be managed in the ICU setting, including airway support where there is reduced consciousness. In severe cases of ICANS, with features of cerebral oedema, supportive measures to manage raised intracranial pressure (ICP), including use of ICP monitors, targeting cerebral perfusion pressure and hyperosmolar therapy are advocated by some groups.[10, 44]

## Other neurological complications of CAR-T

Aside from ICANS, recipients of CAR-T cells, there have also been case reports of PRES [47], and progressive multifocal leukoencephalopathy after CAR-T therapy [48]. Intracranial haemorrhage has also been rarely observed [8, 11]. Unravelling causation in these cases is challenging given the contributions of underlying malignancy and prior conditioning therapies. The incidence of infection within the first 28 days of CAR-T therapy was almost 25% in one case series of 133 patients, although the rate and nature of infections seem to be in line with other salvage chemoimmunotherapy approaches for haematological malignancy [49]. Infections were commonly bacterial or viral respiratory illness, but also included the finding of EBV or CMV in the CSF, and occasionally disseminated fungal infection. All patients commencing CAR-T therapy should routinely receive prophylaxis for herpes infection and prophylaxis for Pneumocystis pneumonia, but anti-bacterial and systemic anti-fungal prophylaxis are not routinely recommended [44].

## Conclusions

CD19 CAR-T therapies now provide an effective treatment for previously refractory B cell malignancies. However, ICANS is a common complication, which can be serious, and rarely fatal. Regular evaluation of CAR-T recipients is required during the first 14 days using validated scoring tools. Treatment of ICANS is largely supportive, including vigilant exclusion of infection and adequate treatment of seizures. Corticosteroids and intensive care support are the mainstay of treatment for higher grades of ICANS. Novel therapies such as siltuximab and anakinra warrant further study and data on long-term neurological outcomes following CAR-T are awaited.
